# Supplementary material for: Identifying Early Risk Factors for Postoperative Pulmonary Complications in Cardiac Surgery Patients
Source: Medicina (Kaunas). 2024 Aug 26;60(9):1398. doi: 10.3390/medicina60091398 (PMC11433804; doi:10.3390/medicina60091398)
Supplement: Supplementary file 1 [file medicina-60-01398-s001.zip › medicina-3153767-supplementary/Table S4.pdf]

# Table S4. Univariate Analysis: Comparison of Risk Factors Across PPC Categories and Control Groups Without PPCs

## Pre-operative risk factors (1)

| PPC types | Right-side PE (n=10) | | p -value | Effect size | Left-side PE (n=45) | | p-value | Effect size | Bilateral PE (n=34) | | p- value | Effect size | Atelectasis (n=44) | | p-value | Effect size | Pulmonary edema (n=25) | | p - value | Effect size | Pneumothorax (n=9) | | p - value | Effect size |
|---|---|---|---|---|---|---|---|---|---|---|---|---|---|---|---|---|---|---|---|---|---|---|---|---|
| Factors | PE | No - PE | | | PE | No - PE | | | PE | No - PE | | | Atelectasis | No - atelectasis | | | Pulmonary edema | No – pulmonary edema | | | Pneumothorax | No - Pneumothorax | | |
| Age (>65 years) | 5 | 177 | 0.403 | | 8 | 174 | 0.324 | | 13 | 169 | **0.015** | **r = 0.1 46** | *30* | 152 | 0.188 | | 14 | 167 | 0.834 | | 5 | 176 | 1.000 | |
| Gender | 6 | 187 | 1.000 | | 33 | 161 | 0.061 | | 25 | 168 | 0.096 | | 45.5% (20) | 64.7% (174) | **0.041** | **r = 0.139** | 13 | 181 | 0.347 | | 6 | 187 | 1.000 | |
| BMI kg/m[2] | 26.75 (24.7;31.45) | 29.1 (26.0;32.41) | 0.297 | | 28.9 (25.4;31.34) | 29.1 (26.1;32.17) | 0.609 | | 28.9(25.4;31.34) | 29.1(26.1;32.17) | 0.440 | | 30.0 (26.7; 32.4) | 28.7 (25.7; 32.1) | 0.179 | | 25.3 (23.4; 28.5) | 29.4 (26.3; 32.45) | **0.001** | **r = 0.170** | 23.7 (19.7;32.3) | 29.2 (26.1;32 | 0.057 | |
| Co-morbidities: | | | | | | | | | | | | | | | | | | | | | | | | |
| Coronary heartdisease (n=192) | 40% (4) | 61% (185) | 0.186 | | 86.7% (39) | 55.8% (150) | 0.001 | **Cr. V = 0.224** | 41.2% (15) | 62.7% (175) | **0.021** | **Cr.V= 0.144** | 52.3% (23 | 166 | 0.178 | | 56% (14) | 60.4% (174) | 0.525 | | 66.7% (6) | 59.9% (182) | 1.000 | |
| Hypertension (n=210) | 50% (5) | 65.5% (199) | 0.308 | | 75.6% (34) | 63.2% (170) | 0.077 | | 67.6% (23) | 64.9% (181) | 0.555 | | 63.6% (28 | 65.2% (176) | 0.729 | | 44% (11) | 66.7% (192) | **0.015** | **Cr.V = 0.14** | 77.8% (7) | 64.5% (196) | 0.723 | |
| Chronic heart failure (n=270) | 90% (9) | 83.2% (253) | 1.000 | | 77.8% (35) | 84.4% (227) | 0.128 | | 79.4% (27) | 84.2% (235) | 0.287 | | 81.8% (36 | 83.7% (226) | 0.478 | | 88% (22) | 83% (239) | 1.000 | | 77.8% (7) | 83.6% (254) | 0.361 | |
| Atrial fibrilation (n=81) | 20% (2) | 24.7% (75) | 0.744 | | 33.3% (15) | 23% (62) | 0.104 | | 29.4% (10) | 23.7% (66) | 0.675 | | 10 | 67 | 0.714 | | 28% (7) | 24.3% (70) | 0.811 | | 11.1% (1) | 25% (76) | 0.457 | |
| DM (n=66) | 30% (3) | 21% (63) | 0.456 | | 26.7% (12) | 20.1% (54) | 0.214 | | 17.6% (6) | 23.7% (59) | 0.663 | | 18.2% (8) | 21.5% (58) | 0.566 | | 8% (2) | 21.9% (63) | 0.124 | | 11.1% (1) | 21.1% (64) | 0.690 | |
| BA (n=8) | 10% (1) | 4.3% (13) | 0.376 | | 4.4% (2) | 4.5% (12) | 0.655 | | 2.9% (1) | 4.3% (12) | 0.567 | | 7 | 57 | 0.431 | | 12% (3) | 3.8% (11) | 0.095 | | 0 | 4.6% (14) | 1.000 | |
| COPD (n=11) | 0 | 2.6% (8) | 1.000 | | 1.4% (2) | 2.2% (6) | 0.331 | | 0 | 8 | 0.605 | | 0 | 14 | 0.233 | | 0 | 2.8% (8) | 0.634 | | 0 | 2.6% (8) | 1.000 | |
| Other lung disease (n=14) | 20% (2) | 4.7% (14) | 0.089 | | 2.2% (1) | 5.4% (15) | 0.291 | | 11.8% (4) | 4.3% (12) | 0.087 | | 0 | 44 | 0.607 | | 12% (3) | 4.5% (13) | 0.379 | | 0 | 5.3% (16) | 1.000 | |
| Chronic renal insufficiency (n=26) | 0 | 7.2% (22) | 1.000 | | 9% (5) | 6.8% (18) | 0.405 | | 5.9% (2) | 7.2% (20) | 1.000 | | 6.8% (3) | 5.9% (16) | 1.000 | | 8% (2) | 6.8% (20) | 0.697 | | 0 | 7.2% (22) | 1.000 | |

Abbreviations: BMI, body mass index; DM, diabetes mellitus; BA, bronchial asthma; COPD, chronic obstructive pulmonary disease.

## Pre-operative risk factors (2)

| PPC types | Right-side PE (n=10) | | p -value | Effect size | Left-side PE (n=45) | | p -value | Effect size | Bilateral PE (n=34) | | p -value | Effect size | Atelectasis (n=44) | | p -value | Effect size | Pulmonary edema (n=25) | | | | Pneumothorax (n=9) | | p -value | Effect size |
|---|---|---|---|---|---|---|---|---|---|---|---|---|---|---|---|---|---|---|---|---|---|---|---|---|
| Factors | PE | No - PE | | | PE | No - PE | | | PE | No - PE | | | Atelectasis | No - atelectasis | | | Pulmonary edema | No – pulmonary edema | | | Pneumothorax | No - Pneumothorax | | |
| SA level before surgery | | | | | | | | | | | | | | | | | | | | | | | | |
| Normal (≥35 g/L) | 90% (n=9) | 87.8% (n=267) | 0.34 | | 82.2% (n=37) | 88.8% (n=239) | 1.000 | | 88.2% (n=30) | 88.2% (n=24) | **0.013** | **Cr.V = 0.2** | 88.6% (n=39) | 87.8% (n=237) | 1.000 | | 88.% (n=22) | 87.8% (n=232) | 1.000 | | 88.9% (n=8) | 87.8% (n=267) | 1.000 | |
| Mild HA (30-35 g/L) | 0 | 0.7% (n=2) | | | 0 | 0.7% (n=2) | | | 5.9%(n=2) | 0.4% (n=1) | | | 0 | 0.7% (n= 2) | | | 0 | 0.7% (n=2) | | | 11.1% (n=1) | 0.7% (n=2) | | |
| Moderate HA (25-30 g/L) | 0 | 0.3% (n=1) | | | 0 | 0.4% (n=1) | | | 0 | 0 | | | 0 | 0 | | | 0 | 0.3% (n=1) | | | 0 | 0.3% (n=1) | | |
| Severe HA (<25 g/L) | 0 | 0 | | | 82.2% (n=37) | 88.8% (n=239) | | | 0 | 0 | | | 0 | 0 | | | 0 | 0 | | | 88.9% (n=8) | 87.8% (n=267) | | |
| EF (%) | 60 (53.5:6 3.5 | 58 (50;60) | 0.367 | | 55 (48.0:60.) | 59 (50.0: 61.0) | **0.035** | **r = 0.138** | 58 (50;60) | 58 (50;61) | 1.000 | | 58.0 (53. 0;60.0) | 58.0 (50.0;6 0.0) | 0.894 | | 56.5 (49;6 0.0) | 58.0 (50.0;6 0.0) | 0.420 | | 58 (50;60) | 8 (50;61) | 0.621 | |
| RVSP (mmHg) | 30 (23.75; 33. | 30 (25;35) | 0.722 | | 30.0 (25.0;32.5) | 30.0 (25.0; 35.0) | | | 30 (25;30) | 30 (25;35) | 1.000 | | 30.0 (25. 0;35.0) | 30.0 (25.0;3 5.0) | 0.289 | | 30.0 (15.0; 35.0) | 30.0 (25.0:3 5.0) | 0.764 | | 40 (35;48.7 | 30 (25;35) | **0.003** | **r = 0.178** |

Abbreviations: SA, serum albumin; HA, hypoalbuminemia; EF, ejection fraction; RSVP, right ventricle systolic pressure.

## Intra-perative risk factors

| PPC types | Right-side PE (n=10) | | p -value | Effect size | Left-side PE (n=45) | | p -value | Effect size | Bilateral PE (n=34) | | p -value | Effect size |
|---|---|---|---|---|---|---|---|---|---|---|---|---|
| Factors | PE | No - PE | | | PE | No - PE | | | PE | No - PE | | |
| PaO2 op before surgery (mmHg) | 160 (140.8; 206.9) | 189.5 (138.5; 188.9) | 0.492 | | 166.0 (133.9: 224.5) | 190.5 (140.8; 250.3) | 0.951 | | 159.0 (120.0; 224.5) | 188.5 (141.0; 249.5) | 0.062 | |
| PaO2 after surgery (mmHg) | 147 (119.7; 184.2) | 160(122. 7:220.2) | 0.821 | | 151.0 (111.5; 180.0) | 163.0 (125.0; 227.0) | **0.019** | **r = 0.133** | 145.0 (108.5; 204.0) | 162.0 (124.75; 225.7) | 0.090 | |
| Difference in Ht level | 9 (5.75; 10) | 8 (5; 10) | 0.686 | | 8.0 (7,0; 10.0) | 8.0 (5.0; 10.0) | 0.179 | | 8.0 (6.0; 10.0) | 8.0 (5.0;10.0) | 0.748 | |
| Surgery tipe | | | | | | | | | | | | |
| Valve surgery (n-146) | 70% ( 7) | 45.7% (139) | 0.198 | | 20% (9) | 51% (137) | **0.001** | **Cr.V = 0.224** | 50% (17) | 46.2% (129) | 0.421 | |
| CABG (n=99) | 20% (2) | 31.9% (97) | 0.513 | | 51% (23) | 28.3% (76) | **0.003** | **Cr.V = 0.171** | 35.3% (12) | 31.2% (87) | 0.697 | |
| Valve surgery + CABG (n=31) | 0 | 10,2% (31) | 0.412 | | 20% (9) | 8.2% (22) | **0.027** | **Cr.V = 0.138** | 0 | 10.8% (30) | **0.026** | **Cr.V= 0.126** |
| Aortic surgery (n=19) | 0 | 6.3% (19) | 1.000 | | 6.7% (3) | 5.9% (16) | 0.744 | | 8.8% (3) | 5.7% (16) | 0.345 | |
| Other | 10% (1) | 5.3% (16) | 0.432 | | 2.2% (1) | 5.9% (16) | 0.483 | | 5.9% (2) | 4% (15) | 1.000 | |
| CPB time (min) | 72.5 (57.70; 102.60) | 90 (73.75;11 0.7) | 0.072 | | 108.0 (76.0 ; 120.5) | 89.0 (72.0; 109.0) | **0.010** | **r = 0.146** | 89.0 (76.0;103.0) | 90.0 (72.75; 111.0) | 0.870 | |
| CPB filling (ml) | 1175.0 (1037.9 ; 1566.0) | 1250.0 (1070 ; 1560.0) | 0.455 | | 1250.0 (1250.0; 1566.5) | 1250.0 (1050.0; 1449.5) | **0.045** | **r = 0.114** | 1250.0 (1125.0; 1564.0) | 1250.0 (1068.9; 147.25) | 0.654 | |
| Fluid Balance After Surgery (ml) | 775.5 (572.0 ; 1250.8) | 700.0 (100.0; 1150.0) | 0.162 | | 750.0 (250.0;1250. 0) | 680.0 (100.0; 1107.0) | 0.991 | | 810.0 (278.5; 1025.0) | 695.0 (100.0 ;1150.0) | 0.809 | |
| Urine output after Surgery (ml) | 950.0 (650.0; 1200 | 1200 (800; 1500 | 0.233 | | 1200 (600; 1450) | 1100 (800; 1600) | 0.529 | | 1300 (700 ;1750) | 1100 (800; 1500) | 0.344 | |
| Blood product transfusion | 80% (8) | 55.3% (168) | 0.195 | | 51.1% (23) | 56.9% (153) | 0.518 | | 64.7% (22) | 55.2% (154) | 0.361 | |
| Intraoperative vasopressor use (n=81) | 30% (3) | 26% (79) | 0.728 | | 24.4% (11) | 26.4% (71) | 0.856 | | 29.4% (10) | 25.8% (72) | 0.677 | |

| PPC types | Atelectasis (n=44) | | p -value | Effect size | Pulmonary edema (n=25) | | p -value | Effect size | Pneumothorax (n=9) | | p -value | Effect size |
|---|---|---|---|---|---|---|---|---|---|---|---|---|
| Factors | Atelectasis | No - atelectasis | | | Pulmonary edema | No – pulmonary edema | | | Pneumo-thorax | No - Pneumothorax | | |
| PaO2 op before surgery (mmHg) | 223.0 (15 3.0; 263.0) | 185.0 (140.0;240. 0) | 0.103 | | 179 (99.5; 242.75) | 189.0 (140.5; 243.5) | 0.404 | | 247.0 (200.8; 280.7) | 186 (140.5; 241.9) | **0.035** | **r = 0.113** |
| PaO2 after surgery (mmHg) | 166.0 (112.0; 223.0) | 159.0 (123.0; 2225.3) | 0.807 | | 142.0 (123.0; 217.0) | 160.0 (122.25; 223.0) | 0.693 | | 219(180.5; 288.9) | 159(121; 222) | 0.075 | |
| Difference in Ht level | 7.0 (4.0 ;10.0) | 8.0 (5.0;10.0) | 0.284 | | 7 (3; 10) | 8 (5; 10) | 0.505 | | 6.5 (0.5;10) | 8 (5; 10) | 0.372 | |
| Surgery tipe | | | | | | | | | | | | |
| Valve surgery (n-146) | 50% (22) | 45.9%(1 24) | 0.745 | | 36% (9) | 47.2% (136) | 0.187 | | 22.2% (2) | 47% (143) | 0.183 | |
| CABG (n=99) | 29.5% (13) | 31.9%(8 6) | 0.862 | | 32%(8) | 31.6%(91) | 1.000 | | 44.4% (4) | 31.3% (95) | 0.472 | |
| Valve surgery + CABG (n=31) | 13.6% (6) | 9.3% (25) | 0.412 | | 8%(2) | 10.1% (29) | 1.000 | | 11.1% (1) | 9.9% (30) | 1.000 | |
| Aortic surgery (n=19) | 6.8% (3) | 5.9% (16) | 1.000 | | 3(12%) | 16 (5.6%) | 0.187 | | 0 | 6.3% (19) | 1.000 | |
| Other | 0 | 6.3% (17) | 0.144 | | 0 | 0 | | | 11.1% (1) | 5.3% (16) | 1.000 | |
| CPB time (min) | 100.0 (76.0;114. 0) | 89.0 (72.0 ;109.0) | 0.192 | | 95.5 (77.25; 114.25) | 89.0 (73.00; 110) | 0.401 | | 85 (60.5;10 | 90 (73; 111) | 0.324 | |
| CPB filling (ml) | 1250.9 (1070.0; 1490.0) | 1250.0 (1 060.0; 1476.5) | 0.931 | | 1250 (1050; 1470) | 1250 (1250.9; 1550.7) | 0.11 1 | | 1250 (1150.8; 1550.9) | 1250 (1068.5 | 0.857 | |
| Fluid Balance After Surgery (ml) | 644.0. (77.0; 900.0) | 7000 (100.0; 1200.0) | 0.203 | | 900 (76.75 ; 1318.75) | 690.0 (100; 1114.0) | 0.409 | | 1200 (729.5; 1318.60 | 700 (100.5; 1025.0) | 0.074 | |
| Urine output after Surgery (ml) | 1200 (100; 1600) | 1200 (700;1500) | 0.136 | | 1100 (800; 1562) | 1150 (800;1500) | 0.963 | | 1200(800; 1800) | 1100(800;15 50) | 0.937 | |
| Blood product transfusion | 68.2% (30 | 54.1% (146) | 0.101 | | 76% (19) | 54.3% (156) | **0.037** | **V.Cr =0.11 9** | 77.8% (7) | 55.3% (168) | 0.308 | |
| Intraoperative vasopressor use (n=81) | 34.1% (15 | 24.8% (67) | 0.267 | | 36% (9) | 25.3% (73) | 0.230 | | 22.2% (2) | 26.3% (80) | 1.000 | |

Abbreviations: Hct, haematocrit, CABG, coronary artery bypass grafting; CPB, cardiopulmonary bypass.

## Post-perative risk factors

| PPC type | Right-side PE (n=10) | | p -value | Effect size | Left-side PE (n=45) | | p -value | Effect size | Bilateral PE (n=34) | | p -value | Effect size | Atelectasis (n=44) | | p -value | Effect size | Pulmonary edema (n=25) | | p -value | Effect size | Pneumothorax (n=9) | | p -value | Effect size |
|---|---|---|---|---|---|---|---|---|---|---|---|---|---|---|---|---|---|---|---|---|---|---|---|---|
| Factors | PE | No - PE | | | PE | No - PE | | | PE | No - PE | | | Atelectasis | No - atelectasis | | | Pulmonary edema | No – pulmonary edema | | | Pneumo-thorax | No - Pneumothorax | | |
| SA level 6h after surgery | | | | | | | | | | | | | | | | | | | | | | | | |
| Normal (≥35 g/L) | 50% (5) | 50.3% (15) | 1.000 | | 55.8% (24) | 70.2% (177) | **0.025** | **Cr.V = 0.145** | 51.6% (n=16) | 70.3% (18) | 0.438 | | 52.3% (30) | 50.0% (135) | 0.538 | | 43.5% (17) | 70.1% (192) | **0.027** | **V.Cr=0 .110** | 66.7% (6) | 49.7% (15) | 0.161 | |
| Mild HA (30-35 g/L) | 40% (4) | 40,8%(n= 14 | | | 41.9%(18) | 26.6% (67) | | | 48.4% (15) | 26.6% (70) | | | 36.4% (20) | 41.5 (112) | | | 52.2% (21) | 26.9% (73) | | | 22.2% (2) | 41.4% (12) | | |
| Moderate HA (25-30 g/L) | 10% (1) | 2%(n=6) | | | 2.3%(1) | 2.4% (6) | | | 0 | 2.7% (7) | | | 4.5% (2) | 1.9% (5) | | | 4.3% (n=1) | 2.2% (6) | | | 11.1% (1) | 2% (6) | | |
| Severe HA (<25 g/L) | 0 | 0.7%(1) | | | 0 | 0.8% (2) | | | 0 | 0.4% (1) | | | 0 | 0.7% (2) | | | 0 | 0.7% (2) | | | 0 | 0.7% (2) | | |
| Serumeproteine level 6h after surgery (g/L) | 55.0 (52.5; 58) | 57.0 (54.0; 60.8) | 0.724 | | 55.0 (51.0; 58.0) | 57.0 (54.0; 60.0) | **0.001** | **r = 0.195** | 57.0 (52.0; 59.75) | 57.0 (54.0; 60.0) | 0.648 | | 56 (53.9; 58.5) | 57 (54; 60) | 0.131 | | 57 (53; 59) | 57 (54; 60) | 0.692 | | 57 (51.5;58.9) | 57 (54; 60) | 0.468 | |
| PaO2 6h after surgery (mmHg) | 136.5 (102.0; 188.8) | 173.5 (128,7; 224.8) | 0.662 | | 160.0 (107.0; 214.0) | 174.0 (130.5;2 20.0) | 0.288 | | 179.0 (126.5; 210.0) | 172.0 (127.0; 220.0) | 0.923 | | 167.0 (111.5; 196.7) | 174.0(129.7 ; 222.5) | 0.060 | | 179.5 (110.5; 224.5) | 172(128.0 ; 218.0) | 0.918 | | 199 (155.5; 255.9) | 172 (126.7; 224.80) | 0.233 | |
| SA level 12h after surgery | | | | | | | | | | | | | | | | | | | | | | | | |
| Normal (≥35 g/L) | 20% (n=2) | 45.1% (128) | 0.620 | | 51.3% (20) | 69.2% (162) | 0.464 | | 55.9% (19) | 58.1% (162) | 0.117 | | 36.4% (16) | 45.6% (12 | 0.004 | **0.321** | 52%(n=13 | 58.3%(18) | 0.244 | | 33.3% (3) | 44.4% (135) | 0.074 | |
| Mild HA (30-35 g/L) | 20% (n=2) | 21.4% (65) | | | 46.2% (18) | 29.1% (68) | | | 20.6% (7) | 28.3% (79) | | | 15.9% (7) | 22.2% (60) | | | 32%(n=8) | 27.1%(n= 78 | | | 11.1% (1) | 21.7% (66) | | |
| Moderate HA (25-30 g/L) | 0 | 0.7% (2) | | | 2.6% (1) | 1.3% (3) | | | 2.9% (1) | 1.4% (4) | | | 4.5% (2) | 0 | | | 4%(n=1) | 1%(n=3) | | | 11.1% (1) | 0.3% (1) | | |
| Severe HA (<25 g/L) | 0 | 0.3% (1) | | | 0 | 0.4% (1) | | | | | | | 2.3% (1) | 0 | | | | 0.3%(n=1) | | | | 0.3% (1) | | |
| PaO2 12h after surgery (mmHg) | 132.5 (118,5; 165.9) | 155(120. 9;203.3) | 0.789 | | 141 (112.0; 190.5) | 156 (120.0; 205.0) | 0.985 | | 160.0 (121.0; 100.5) | 152.0 (119.0; 204.0) | 0.803 | | 157.5 (119.5; 198.5) | 174.0 (129.7; 222.3) | | | 179.5 (122.5; 219.75) | 152(119; 203) | 0.440 | | 189(128;2 4 | 152 (119; 203) | 0.202 | |
| Blood product transfusion | 30% (3) | 29.6% (90) | 1.000 | | 37.8% (17) | 28.3% (76) | 0.381 | | 35.3% (12) | 29%( 81) | 0.299 | | 40.9% (18 | 27.8% (75) | 0.152 | | 48% (12) | 28.1% (81) | **0.040** | **r = 0.125** | 11.1% (1) | 30.3% (92) | 0.444 | |
| Postoperative vasopressor and inotrope use (n=81) | 50% (5) | 35.2% (107) | 0.506 | | 35.6% (16) | 35.7% (96) | 1.000 | | 47.1% (16) | 34.4% (96) | 0.180 | | 40.9% (18) | 34.8% (94) | 0.612 | | 48% (12) | 34.7% (100) | 0.186 | | 55.6% (5) | 35.2% (107) | 0.296 | |
| First day fluid balance (ml) | 500 (0.0; 847.9) | 0.0(- 850.0; 680) | 0.086 | | 0 (-1184.5; 690.9) | 0.0 (- 773.0; 647.8) | 0.493 | | 0.0 (- 1211.75; 610.0) | 0.0 (- 805.0; 660.0) | 0.648 | | 0.0 (- 830.0; 510.0) | 0.0 (- 840.0; 684.5) ) | 0.824 | | 380 (0.0; 962.5) | 0 (-830; 640) | 0.062 | | 0 (-1819.5; 670.0) | 0 (-810;644) | 0.928 | |
| Surgery day ICU urin output (ml) | 2975.0 (1975.9 0) | 2900 (2300.0) | 0.949 | | 3000.0 (2450.0; 4400) | 50.0 (2300.00; 3730.80) | 0.506 | | 3750.0 (2625.0; 4625.0) | 2850.0 (2300; 3700.0) | **0.010** | **r=0.1** | 2800 (21 00; 3900) | 2900 (2337.5; 3750.0) | 0.769 | | 2505 (2175.0;37 30.0) | 2950 (2457.3 ;38 80.7) | 0.137 | | 3370(1950 .9; 4500.9) | 2900 (2300; 3800.9 | 0.532 | |
| Time to extubation (h) | 4.5(3.0; 5.6) | 6.5 (5.0; 8.0) | 0.004 | | 6.25 (4.87; 8.0) | 6.5 (5.0; 8.0) | 0.563 | | 6.5 (5.1; 7.37) | 6.5(5.0; 8.0) | 0.742 | | 6.0(4.5; 8.0) | 6.5(5.0;8.0) | 0.294 | | 5.0 (4.5; 8.0) | 6.7 (5.0; 8.8) | 0.294 | | 6 (4.75; 10.2) | 6,5(5;8) | 0.760 | |
| Days at ICU | 1(1; 1) | 1(1; 2) | 0.224 | | 1 (1; 2) | 1 (1; 1) | 0.455 | | 1 (1; 2) | 1(1; 1) | **0.009** | **r=0.1** | 1 (1; 2) | 1(1; 1) | 0.071 | | 1 (1; 3.5) | 1(1; 1) | **0.003** | **r=0.21** | 1 (1;1.5) | 1 (1; 2) | 0.68 | |
| Days at hospital | 13.6 (10 ; 14) | 11.0 (9.0; 14.9) | 0.302 | | 12 (9; 18) | 11 (9; 14) | 0.108 | | 10.0 (8.0; 14.0) | 11.0 (9.0; 14.0) | 0.334 | | 12 (10; 15) | 11 (9; 14) | 0.084 | | 12 (10; 14) | 11 (9; 14) | 0.340 | | 13 (8; 14) | 11(9; 14) | 0.915 | |

Abbreviations: SA, serum albumin; HA, hypoalbuminemia; ICU, intensive care unit.
