# Supplementary material for: Identifying Early Risk Factors for Postoperative Pulmonary Complications in Cardiac Surgery Patients
Source: Medicina (Kaunas). 2024 Aug 26;60(9):1398. doi: 10.3390/medicina60091398 (PMC11433804; doi:10.3390/medicina60091398)
Supplement: Supplementary file 1 [file medicina-60-01398-s001.zip › medicina-3153767-supplementary/Tables S1-S3.pdf]

Table S1. Univariate Analysis of Risk Factors for Left-Sided Pleural Effusion

Variables	Left-Sided Pleural Effusion (n=45)	Control Group (n=269)	p-value	Effect Size
EF, %	55 (48.0:60.0)	59 (50.0:61.0)	0.035	r=0.138
PaO2 befor surgery (mmHg)	151.0 (111.5; 180.0)	163.0 (125.0; 227.0)	0.019	r=0.133
Valve surgery (n=146)	20% (9)	51% (137)	0.001	Cr.V=0.220
CABG (n=99)	51% (23)	28.3% (76)	0.003	Cr.V=0.171
Valve surgery + CABG (n=31)	20% (9)	8.2% (22)	0.027	Cr.V=0.138
CPB time, min	108.0 (76.0:120.5)	89.0 (72.0:109.0)	0.010	r=0.146
CPB priming volume, ml	1250.0 (1250.0; 1566.5)	1250.0 (1050.0; 1449.5)	0.045	r=0.114
Postoperative SA level				
Normal (≥35 g/L)	55.8% (n=24)	70.2% (n=177)	0.025	V=0.145
Mild hypoalbuminemia (30-35 g/L)	41.9% (n=18)	26.6% (n=67)		
Moderate hypoalbuminemia (25-30 g/L)	2.3% (n=1)	2.4% (n=6)		
Severe hypoalbuminemia (<25 g/L)	0	0.8% (n=2)		
Postoperative serum protein level (g/L)	55.0 (51.0:58.0)	57.0 (54.0; 60.0)	0.001	r=0.195

Abbreviations: EF, ejection fraction; CABG, coronary artery bypass grafting; CPB, cardiopulmonary bypass; SA, serum albumin.

Table S2. Univariate Analysis of Risk Factors for Bilateral Pleural Effusion

**Variable**	**Bilateral Pleural Effusion (n=13)**	**Control group (n=269)**	**p-value**	**Effect size**
Age (>65 years)	38.2% (13)	60.4% (169)	0.015	r=0.146
Valve Surgery + CABG	0	10.8% (30)	0.036	Cr.V=0.114
Urine output, ml	3750.0 (2625.0; 4625.0)	2850.0 (2300; 3700.0)	0.010	r=0.146
ICU days	1 (1; 2)	1 (1; 1)	0.009	r=0.149

Abbreviations: Coronary artery bypass grafting; CPB; ICU, intensive care unit.

Table S3. Univariate Analysis of Risk Factors for Pulmonary edema

Variable	Pulmonary edema (n=25)	Control group (n=289)	p-value	Effect size
BMI, (kg/m2)	25.3 (23.4; 28.5)	29.4 (26.3; 32.45)	0.001	r=0.172
Intraoperative transfusions	76% (19)	54.3% (156)	0.037	Cr.V=0.119
Postoperative SA level				
Normal (≥35 g/L)	43.5% (n=10)	70.1% (n=190)	0.027	Cr.V=0.164
Mild hypoalbuminemia (30-35 g/L)	52.2% (n=12)	26.9% (n=73)		
Moderate hypoalbuminemia (25-30 g/L)	4.3% (n=1)	2.2% (n=6)		
Severe hypoalbuminemia (<25 g/L)	0	0.7% (n=2)		
Postoperative serum protein level (g/L)	55.0 (51.0:58.0)	57.0 (54.0; 60.0)	0.001	r=0.195
Postoperative transfusions	48% (12)	28.1% (81)	0.040	Cr.V=0.125
ICU Days	1 (1; 3.5)	1 (1; 1)	0.003	r=0.219

Abbreviations: BMI, body mass index; SA, serum albumin; ICU, intensive care unit.
